# Effects of Rumen-Protected Niacin on Dry Matter Intake, Milk Production, Apparent Total Tract Digestibility, and Faecal Bacterial Community in Multiparous Holstein Dairy Cow during the Postpartum Period

**DOI:** 10.3390/ani11030617

**Published:** 2021-02-26

**Authors:** Naren Gaowa, Xiaoming Zhang, Huanxu Li, Yajing Wang, Jun Zhang, Yangyi Hao, Zhijun Cao, Shengli Li

**Affiliations:** 1 State Key Laboratory of Animal Nutrition, Beijing Engineering Technology Research Center of Raw Milk Quality and Safety Control, College of Animal Science and Technology, China Agricultural University, Beijing 100193, China; narengaowa@cau.edu.cn (N.G.); xiaomingzh@cau.edu.cn (X.Z.); yajingwang_cau@163.com (Y.W.); B20193040338@cau.edu.cn (Y.H.); caozhijun@cau.edu.cn (Z.C.); 2 Beijing Oriental Kingherd Biotechnology Company, Beijing 100069, China; luxsay@sohu.com; 3 College of Animal Science and Technology, Northwest A&F University, Yangling 712100, China; Jzhang0701@nwafu.edu.cn

**Keywords:** rumen-protected niacin, dry matter intake, milk production, multiparous Holstein dairy cow, postpartum period

## Abstract

**Simple Summary:**

The main challenge faced by cows during the first three weeks after calving is the sudden increase in nutrient demand for milk production while dry matter intake and nutrient supply lag. Although metabolic hormone changes are a normal adaptive process in high yielding cows, failure to adapt to this challenge can lead to disease states and affect production and reproductive efficiency. Therefore, a smooth transition from prepartum to postpartum period for optimal dairy cow health and performance. Whether rumen-protected niacin supplementation could be a useful additive was controversial. This work tested whether the widespread use of rumen-protected niacin in multiparous Holstein dairy cows could be justified.

**Abstract:**

Extensive studies about rumen-protected niacin (RPN) supplementation on dairy cows in early-lactation have been done, but the effects of RPN on changes in dry matter intake (DMI), milk production, feed digestibility, and fecal bacterial community were conflicting. The aim of this study was to investigate them affected by RPN in postpartum cows. Multiparous Holstein dairy cows (*n* = 12, parity = 3.5 ± 0.5, body weights = 740 ± 28 kg) were divided into two groups supplemented with either 0 (CON) or 20 g/d RPN (RPN). Our results showed that RPN supplementation increased DMI and milk production of cows during the first three weeks after calving (*p* < 0.05). The concentrations of neuropeptide Y and orexin A were significantly higher in RPN group than that in the CON group during postpartum period (*p* < 0.05). The apparent total-tract digestibility of nutrients was similar between the CON and RPN groups at 2 weeks after calving (*p* > 0.05). The 16S rRNA gene sequencing analysis showed that RPN had no impact on the alpha and beta diversity, although 4 genera were changed in cow feces at 14 days after calving. Overall, 20 g/d RPN added to the diet could improve DMI and milk yield up to two weeks after calving with little influence on feed digestibility.

## 1. Introduction

As for farmed animals, the health and biological functioning of dairy cattle are often prioritized. Pregnancy and postpartum are recognized as inducing remarkable physiological and metabolic adaptations in dairy cows essential for a good reproductive performance and to ensure the suitable development of the fetus and to provide adequate substrates that are needed in utero and following birth [1,2,3]. Despite the action of homeostatic mechanisms to maintain blood parameters within physiologic levels, changes in metabolites and hormones occur as a result of increased metabolic demands in lactating animals. These changes are not necessarily indicative of diseases but make animals physiologically unstable and more susceptible to a number of metabolic diseases at this stage than during other life periods compromising productivity [4]. These repercussions highlight the need for a smooth transition from prepartum to postpartum period for optimal dairy cow health and performance. The improvement of diet composition becomes a key factor to improve the health status and welfare of animals [5], as well as to enhance productivity in livestock [6,7,8].

Niacin or nicotinic acid (NA) is a B vitamin and precursor of nicotinamide adenine dinucleotide phosphate and nicotinamide adenine dinucleotide, which are key coenzymes in many enzymatic reactions in intermediary metabolism, including lipid and glucose metabolism [9,10]. In dairy cows, NA can be synthesized de novo by the rumen microbiome [11]. However, synthesis of NA is an energy-demanding process and, in situ, rumen bacteria may synthesize only what they require for growth and function [12,13,14]. In recent years, administration of dosages of NA, either in rumen-protected form or via the abomasum to reduce or avoid ruminal degradation, have been evaluated in dairy cow studies [10,15,16,17,18]. Most of these focus on the anti-lipolytic effects of NA, which may reduce blood non-esterified fatty acids (NEFA) [10] or β-hydroxy butyric acid (BHBA) [17,19]. Although the requirements of niacin are still unknown, high doses of it may have beneficial effects under certain metabolic conditions. The observations on many farms in China suggest that postparturient cows consuming 20 g/d of rumen-protected niacin (RPN) had a stronger desire to feed. Whether RPN improves postpartum DMI in cows by affecting blood levels of appetite-related hormones is something that needs to be studied with more data.

Feed intake in dairy cows may be controlled by many hormones such as neuropeptide Y (NPY) [20,21] and orexin A (OXA) [22]. NPY is involved in a variety of physiological and homeostatic processes in the central and peripheral nervous systems and induces increased food intake [23,24]. Orexins are hypothalamic neuropeptides including OXA / orexin 1 and orexin B / orexin 2 [25]. They are derived from common precursor peptides and bind to the type 1 orexin receptor (OX1R) and type 2 orexin receptor (OX2R), respectively. The OX1R has an important role in the regulation of food seeking, while the OX2R is a major player in the regulation of wakefulness [26,27]. In other words, food-seeking can be motivated by increasing OXA [28,29]. Moreover, the nutrient processing [30] and production performance [31] of host animals were related to their gastrointestinal microbiology. The microbial composition of feces is also related to feeding management in cattle [32]. However, few niacin studies mentioned the changes of microbial composition in feces. Therefore, the aim of this work was to determine the effect of RPN on feeding performance (DMI, NPY, and OXA), milk production, and feed digestibility (apparent total tract digestibility and the microbial composition of feces) in multiparous Holstein dairy cows during the postpartum period.

## 2. Materials and Methods 

### 2.1. Ethics Statement

Throughout the experiment, all animals involved in this study were managed according to the herd standard protocol at the Sunlon Livestock Jinyindao Farm (Daxing County, Beijing, China). All animal protocols were reviewed and approved by China Agricultural University, Institutional Animal Care and Use Committee (Beijing, China, permit No. AW10012020-2-1).

### 2.2. Experimental Design and Sample Collection

A total of 12 multiparous Holstein dairy cows were divided into two groups with diet supplemented with either 0 (CON) or 20 g/day RPN (RPN), each group contained three 3rd parity and three 4th parity cows, respectively. The expected calving date (difference less than two weeks) and cumulative milk yield (total milk yield of 9210 to 10,870 kg for 305 days of the previous lactation cycle) of the cows enrolled in the trial were similar. The body weight (734.67 ± 29.95 and 745.33 ± 27.33 in CON, and RPN group, respectively), ages (57.83 ± 6.74 and 57.0 ± 6.84 months in CON and RPN group respectively), and body condition score were not different (*p* > 0.10) among groups at the beginning of the study. The cows were dried off on d 60 before calving and housed in an individual pen that includes comfortable bedding and dry lot. During the trial, the total mixed ration (TMR) of the dry cow and lactating cow was provided twice daily at 7:30 and 14:00. During the close-up period, the prepartum TMR was provided once daily at 14:00. The RPN (Anynew N, Beijing Oriental Kingherd Biotechnology Company) included 55% of pure niacin, and the 12-h ruminal stability was 96%. RPN supplementation was from 49 days before to 21 days after calving. In each day morning before feeding, cows in the RPN group were fed a mixture of 20 g of RPN and 1 kg of fresh TMR, where a small amount of molasses was used to combine them together. For the CON group, cattle were fed a mixture of 1 kg of fresh TMR and a small amount of molasses. The composition of the basal diet and the proportion of nutrients are provided in Appendix A. 

All the samples were taken individually from each cow besides TMR samples. Samples of TMR were collected twice a week during the experiment to monitor the dry matter (DM) content. Daily feed intake was monitored by Roughage Intake Control System (RFID, Zhenghong Company, Shanghai, China), a system that can identify the cattle ID before opening the trough and measure feed intake by collecting the feed weight before and after eating. Milk production was recorded every day after milking. Milk samples were collected and mixed with potassium dichromate stored at 4 °C for no more than 24 h on days 7, 14, and 21 after calving. Blood samples were collected from the coccygeal vein into 5 mL evacuated serum tubes (Vacutainer; Becton Dickinson, Franklin Lakes, Nanjing, China) on days 1, 3, 7, 14, and 21 at 6:00 a.m. The sera were obtained by centrifuging (3500× *g* at 4 °C for 15 min) and stored at −20 °C for further analysis of NPY, OXA, NEFA, and BHBA. Blood samples from all time points would be uniformly tested once the animal experiment was done. Faecal samples were collected three times a day, eight hours apart on days 13, 14, and 15. All the samples from each cow were mixed with tartaric acid and homogenized to create a single bulk sample, dried at 65 °C to a constant weight in an air-forced oven, and ground for further chemical analysis. To coincide with sampling times for feed digestibility, 2 g faecal samples were sealed in 2 mL conical tubes at two weeks after calving (morning of day 14) and frozen in liquid nitrogen, then stored at −80 °C until genomic DNA was extracted. However, one of the 2 g faecal samples in the CON group lost during the transfer of samples from farm to laboratory.

### 2.3. Analytical Procedures

The concentrations of DM, crude protein (CP), ether extract (EE), ash, calcium, and phosphorus in basal diets and feces were analyzed according to the methods established by the Association of Official Analytical Chemists (AOAC) [33]. Neutral detergent fiber (NDF) and acid detergent fiber (ADF) analyses followed the protocols described by Hao et al. [34]. Alpha-Amylase (ANKOM Technology Co., Ltd., Macedon, NY, USA) was added for NDF analysis. Acid-insoluble ash was employed as a marker of internal tract digestibility to analyze the apparent total tract digestibility [35].

Protein and fat in milk were analyzed in a DHI Testing Center (Beijing, China) by using an automated near-infrared milk analyzer (CombiFoss FT+; Foss Electric, Hillerød, Denmark). Blood serum NEFA (No. E030-1-1) and BHBA (No. A042-1-1) were analyzed on a Hitachi 7600 automated biochemistry analyzer (Hitachi Co. Ltd., Tokyo, Japan) using kits from Nanjing Jiancheng Bioengineering Institute (Nanjing, China). The ELISA method was used to determine the NPY (Bovine neuropeptide Y kit, SRB-T-85362) and OXA (Bovine Orexin A ELISA kit, 201-04-3851) kits from Shanghai Horabio Biotechnology Co., Ltd. (Shanghai, China).

### 2.4. Faecal Bacterial Community Analytical Procedure

The main protocol of faecal bacterial DNA extraction has followed the procedure described by Hao et al. [34]. 16S rRNA gene amplicon preparation and sequencing were as same as the description in the previous study [36]. Briefly, DNAs were extracted using HiPure Stool DNA Kits (Magen, Guangzhou, China). The qualities of the DNA were appraised using a NanoDrop ND-1000 Spectrophotometer (NanoDrop Technologies, Wilmington, DE, USA). The extracted DNA was amplified by PCR with the KAPA HiFi Hotstart ReadyMix PCR kit (KAPA Biosystems, Wilmington, MA, USA). The V3–V4 region of the bacterial 16S rRNA gene was amplified using primers F341 (5′-ACTCCTACGGGRSGCAGCAG-3′) and R806 (5′-GGACTACVVGGGTATCTAATC-3′) [37]. The amplicons were gathered from 2% agarose gels and purified with a QIAquick PCR Purification Kit (Qiagen, Hilden, Germany). Purified DNAs were re-quantified using an Agilent DNA 1000 Kit (Agilent Technologies, Waldbronn, Germany). Library quality was assessed on a Qubit 2.0 Fluorometer (Life Technologies, Grand Island, NY, USA). Sequencing library preparation was done using NEBNext ultra DNA sample preparation kit (New England Biolabs Inc., Ipswich, MA, USA). Then, reads of approximately 250–300 bp paired-end were sequenced on the Illumina MiSeq platform.

### 2.5. Bioinformatics and Statistical Analyses

16S rRNA microbiota data analysis has followed the pipeline published in the author’s study [36]. Briefly, the quality control of raw data was done by FastQC. Concatenated sequences were detected using USEARCH. Sequence analyses and alpha diversity were performed using QIIME pipeline (version 1.5.0) [38]. Beta diversity was measured according to weighted UniFrac distances and displayed using principal coordinate analysis (PCoA) based on ‘vegan’ package in R. Kruskal-Wallis non-parametric test was employed to compare the differences of genera abundance between RPN and CON groups. The 16S rRNA raw reads obtained from feces of cows were submitted to NCBI with project number PRJNA682766.

The weekly means were calculated from daily records of DMI and milk production prior to statistical analyses. Analyses of variance of the data were performed using PROC MIXED of SAS 9.2 (SAS Institute Inc., Cary, NC, USA). The model included the fixed effects of treat, time, treat × time interaction, and the random effect of individual animals. Effects of treatment on DMI, NPY, OXA, NEFA, BHBA, and milk production for each time point were tested using one-way ANOVA. For all variables, significant treatment and interaction effects were noted at *p* ≤ 0.05. 

## 3. Results

### 3.1. Effects of RPN on Production Performance in Multiparous Holstein Dairy Cows in Postpartum Period

As shown in Table 1, DMI was increased in RPN compared to CON within three weeks before calving (*p* < 0.05). DMI, the concentration of NPY and OXA, milk production, and milk protein yield were higher in RPN than that in CON (*p* ≤ 0.001) during the first three weeks after calving. Yet, the concentration of the milk protein and fat, production of energy-corrected milk and milk fat were not changed significantly by RPN supplement. The changes of sera NEFA were affected by not only treatment but also the time points. The concentration of BHBA in blood was significantly increased by treatment. However, it was affected by the interaction of RPN and time. 

**Figure 1:**
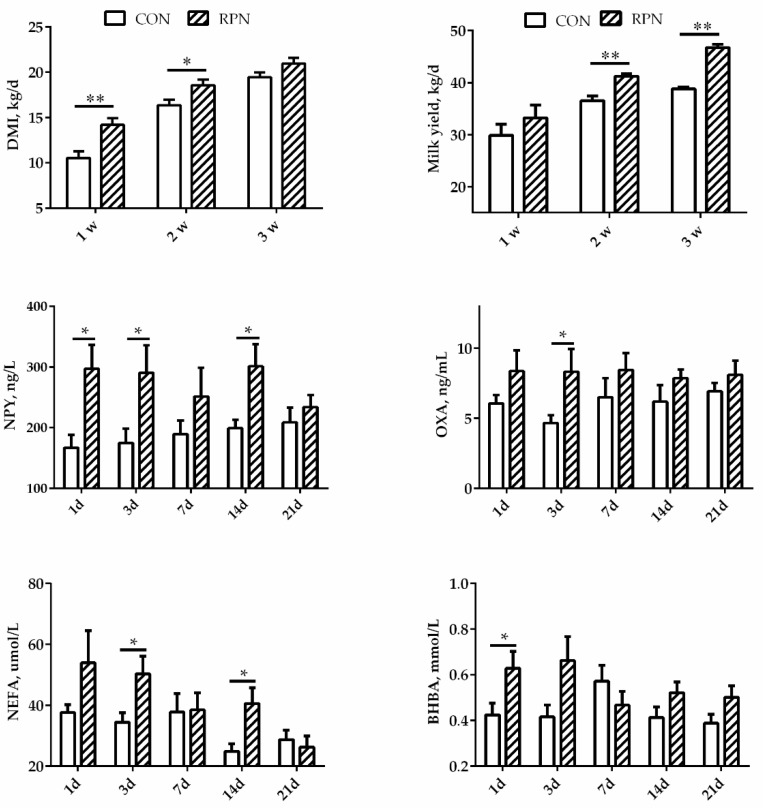
Effects of rumen-protected niacin on dry matter intake (DMI), neuropeptide Y (NPY), orexin A (OXA), milk yield, and the concentrations of non-esterified fatty acids (NEFA) and β-hydroxy butyric acid (BHBA) in multiparous Holstein dairy cow at different time points. CON: Control; RPN: rumen-protected niacin; *: *p* < 0.05; **: *p* < 0.01.

As shown in Figure 1, DMI increased significantly in cows in RPN group during the first two weeks after calving (*p* < 0.05) and kept increasing until the third week (*p* = 0.068). Milk yield increased significantly in RPN group during the second and third weeks after calving (*p* < 0.01) though no affected in the first week (*p* = 0.317). The concentration of NPY in blood was significantly higher on days 1, 3, and 14 in RPN compared with CON. The concentration of OXA was significantly higher on day 3 in RPN than that in CON (*p* < 0.05). The concentration of NEFA in blood was significantly higher on days 3 and 14 after calving. The effects of the RPN on the concentration of BHBA were not significant on days 3, 7, 14, and 21, although BHBA was significantly higher on the first day after calving in RPN group (*p* < 0.05).

### 3.2. Effects of RPN on the Apparent Total-Tract Digestibility of Nutrients and Faecal Microbial Community in Multiparous Holstein Dairy Cow at the Second Weeks after Calving

**Figure 2:**
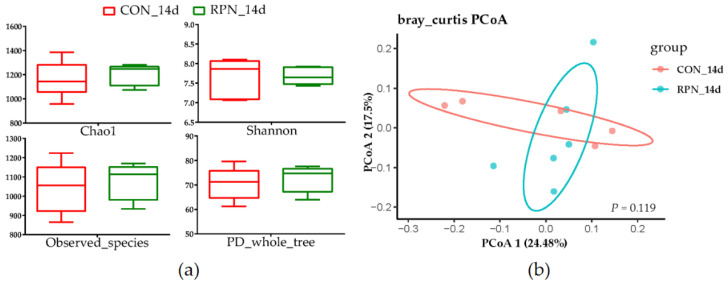
Alpha; and (**a**) beta; (**b**) diversity of the faecal microbial community in control (CON_14d) and rumen-protected niacin (RPN_14d) groups.

No effects of RPN on the apparent total-tract digestibility of NDF, ADF, EE, CP, OM, and DM (Table 2, *p* > 0.05), alpha and beta diversity of the faecal microbial community (Figure 2, *p* > 0.05) were observed in multiparous Holstein dairy cow at the second week after calving. The abundances of most genera were not significantly different (Supplementary Table S1). However, the genera of *Eubacterium_nodatum_group*, *Dorea*, *Mycoplasma*, and *Escherichia-Shigella* in feces were significantly different between RPN and Con groups. The abundances of *Eubacterium_nodatum_group*, *Dorea*, and *Escherichia-Shigella* were higher whereas the genus of *Mycoplasma* was lower in feces of RPN compared with that of CON (Table 3, *p* < 0.05).

## 4. Discussion

DMI results vary widely in niacin supplementation studies. Rumen unprotected niacin (0, 16, 32, or 48 g/d) resulted in linear decreases in DMI [39]. Rumen-protected niacin had no effect on postpartum DMI in many studies [9,15,40,41]. In contrast, a 3.6% increase in DMI was observed when feeding 12 g/d NA [42]. In our study, supplementation of 20 g RPN (NA ≈ 11 g) increased DMI as well. The increased blood concentration of NPY suggests that the appetite of cows supplied with 20 g RPN had been strengthened during the postpartum period. Although the blood concentration of OXA did not change significantly, the value was numerically higher in the RPN group when compared with the CON group during the postpartum period. In this study, the differences in feed intake corresponded to differences in intake-related hormones, which further illustrated that 20 g/d RPN supplementation can enhance the feeding performance of multiparous Holstein dairy cows during the postpartum period. The digestibility of the main nutrients did not differ between the two groups, which is in agreement with previous work [14,43]. These results highlight the potential of RPN to promote nutrient intake during the postpartum period via increased DMI.

Changes in milk production and DMI were consistent in our study (improved 5.32 and 3.22 kg/d respectively). However, other studies found that diet supplemented with a low dosage of RPN (12 g/d) [9,40] or non-protected NA (24 g/d) [44,45], and high dosages of RPN (55 g/d) [46] and NA (120 g/d) [17] did not change milk production. Notably, different from these studies, we treated the cows with RPN for a longer period (beginning on day 49 before calving and ending on day 21 after calving). Whereas the earliest dosing day of studies mentioned above was 21 days before calving. Interestingly, a decline in serum NEFA or BHBA was reported in the same studies in which milk production did not change [9,17,47]. In contrast, milk production and DMI increased with higher serum NEFA and BHBA in cows supplemented with 20 g/d RPN in our study. Previously, serum NEFA concentrations have been associated with ECM [9]. In this work, although ECM was not significantly different among groups, expanded serum NEFA concentration was consistent with higher ECM in the RPN group. The rise of NEFA and BHBA through niacin supply suggested that a higher degree of fat mobilization despite an increased feed intake. However, BHBA and NEFA concentrations of all cows in the present study were at a lower range than was reported by other studies for early lactation cows [47,48,49]. The concentration of NEFA ≥ 0.70 mmol/L is a potential marker for postpartum health problems [50]. Concentrations of BHBA ≥ 1.2 mmol/L are used to define hyperketonemia [51]. Yet, the concentrations of BHBA and NEFA in cows included in our experiment were much lower than 1.2 mmol/L, and 0.70 mmol/L, respectively. On the other hand, the cows were 3rd and 4th parity and were treated with 20 g/d RPN in the transition period of their preceding lactation period (2nd or 3rd lactation). This may help relieve the metabolic stress (e.g., increased serum NEFA and BHBA as mentioned in other studies) during the transition period and maintain homeostatic metabolism after calving.

Gut microbes are closely related to host health [52]. Feces are an easy-to-collect sample that can represent the intestinal microorganisms and illustrate the changes in their microbial communities [53,54,55,56]. As the predominant genus in the feces of cattle, *Ruminococcus* was a fiber utilizer [57] and *Bacteroides* was a polysaccharides utilizer [58]. However, no significant differences were found in the abundance of *Ruminococcus* and *Bacteroides* between the two groups in our study. These findings were consistent with no difference in NDF and ADF digestibility. Genera of *Eubacterium_nodatum_group*, *Dorea*, and *Escherichia-Shigella* were conventional in cattle feces and reported in many studies [55,56,59,60]. Many opportunistic pathogens belong to the genera *Eubacterium_nodatum_group* and *Escherichia-Shigella* [61]. Genus of *Eubacterium_nodatum_group* could utilize lysine and arginine to produce acetate and butyrate [62], which will result in a relative increase in many pathogens [63]. However, *Escherichia-Shigella* was the dominant genus in the uterus of healthy cows compared with the cows with metritis [64]. Moreover, the abundances of *Eubacterium_nodatum_group* and *Escherichia-Shigella* in this study were very low (about 0.3% in total in RPN group). Therefore, changes in their relative abundance may not be of biological relevance. *Dorea* is a gram-positive bacterial genus from the family *Lachnospiraceae* [65]. The abundance of *Dorea* in the feces of dairy cows increased when probiotics administration improved the host health [65] and the milk production [55]. Similarly, in the previous study, cows fed diets supplemented with RPN not only improved the milk production but also the abundance of *Dorea* in feces. These indicated that host healthy conditions benefit the growth of *Dorea*. *Mycoplasma* was isolated from bovine feces as early as 1975 [66]. Several species in the *Mycoplasma* genus are pathogenic in cattle [67,68]. The lower abundance of it in RPN cows may be another healthy sign illustrating that RPN can help multiparous Holstein dairy cows to smoothly transfer from prepartum to the postpartum period.

## 5. Conclusions

Our work provides a reference for the use of RPN during the transition period to improve DMI and milk production performance in multiparous Holstein dairy cows in early lactation. Dietary supplementation with 20 g/d RPN could improve DMI via increased intake-related hormones, NPY and OXA, but had little influence on feed digestibility and faecal bacterial community. Dietary supplementation with 20 g/d RPN could also significantly improve milk production without significant impact on ECM or negative impacts on serum NEFA and BHBA. Further research is needed to determine whether RPN has a cumulative effect on multiparous Holstein dairy cows in subsequent transition periods. Moreover, further research is deserving whether the above-mentioned effects are physiological or pharmacological. 

## Figures and Tables

**Table 1:** Effects of rumen-protected niacin on feeding performance, milk performance, and serum concentration of metabolites in multiparous Holstein dairy cow in the first three weeks after calving.

Item	Treatment		SEM	*p*-Value		
	CON	RPN		Treat	Time	Treat × Time
Feeding performance						
DMI^pre^, kg/d	11.64	12.97	0.278	0.025	<0.001	0.862
DMI, kg/d	14.89	18.11	0.364	<0.001	<0.001	0.281
NPY, ng/L	188.91	274.04	11.095	<0.001	0.855	0.471
OXA, ng/mL	5.85	8.22	0.354	0.001	0.726	0.803
Yield, kg/d						
Milk yield	35.08	40.4	1.000	<0.001	<0.001	0.267
Milk protein	1.28	1.49	0.033	0.001	0.628	0.529
Milk fat	1.35	1.33	0.082	0.926	0.307	0.907
ECM	32.75	35.26	1.448	0.257	0.028	0.655
Milk composition						
Milk fat, %	3.74	3.17	0.201	0.189	0.831	0.809
Milk protein, %	3.61	3.58	0.072	0.864	<0.001	0.653
Serum concentration of metabolites						
NEFA, umol/L	32.56	41.63	1.945	0.007	0.007	0.176
BHBA, mmol/L	0.44	0.55	0.021	0.003	0.357	0.048

ECM, kg/d = 12.82 × fat yield + 7.13 × protein yield + 0.323 × milk yield. ECM: Energy-corrected milk; DMI^pre^: dry matter intake of three weeks before calving; DMI: dry matter intake of the first three weeks after calving; NPY: neuropeptide Y; OXA: orexin A; NEFA: non-esterified fatty acids; BHBA: β-hydroxy butyric acid; CON: Control; RPN: rumen-protected niacin; SEM: standard error of the mean.

**Table 2:** Effects of rumen-protected niacin on the apparent total-tract digestibility of nutrients in multiparous Holstein dairy cow at 2 weeks after calving.

Item	CON	RPN	*p*-Value
NDF, %	60.79 ± 10.22	64.20 ± 5.94	0.373
ADF, %	58.69 ± 9.86	61.13 ± 6.09	0.513
EE, %	85.77 ± 4.47	85.75 ± 4.76	0.993
CP, %	77.51 ± 5.20	79.29 ± 4.02	0.439
OM, %	77.06 ± 4.37	79.34 ± 3.77	0.229
DM, %	77.34 ± 3.99	79.20 ± 3.49	0.281

CON: Control; RPN: rumen-protected niacin; DM: dry matter; OM: organic matter; CP: crude protein; NDF: neutral detergent fiber; ADF: acid detergent fiber.

**Table 3:** Significantly different genera between rumen-protected niacin and control groups in the second week (on d 14) after calving.

Genus	Abundance (%, Means ± SE)		*p*-Value
	RPN	CON	
*Eubacterium_nodatum_group*	0.2833 ± 0.1143	0.1569 ± 0.0745	0.0285
*Dorea*	0.3801 ± 0.1737	0.1971 ± 0.0556	0.0353
*Mycoplasma*	0.0013 ± 0.0016	0.0088 ± 0.0082	0.0379
*Escherichia-Shigella*	0.0255 ± 0.0185	0.0064 ± 0.0061	0.0441

CON: Control; RPN: rumen-protected niacin; SE: standard error.

## Data Availability

The 16S rRNA raw reads obtained from feces of cows were submitted to NCBI with project number PRJNA682766.

## Supplementary Materials

The following is available online at https://www.mdpi.com/2076-2615/11/3/617/s1, Supplementary Table S1: The abundance of genera between RPN and CON groups.

## Appendix A

**Table A1:** The Ingredient and Nutrient Levels of Experiment Diets.

Ingredient, % DM	Groups		
	Close-up	Far-off	Fresh Cow
Corn silage	56.2	61.2	55.8
Chinese wildrye	11.7	- -	- -
Oat	16.4	19.6	2.50
Alfalfa	- -	- -	6.30
DDGS ^1^	- -	2.70	1.60
Sprayed corn husk	9.50	6.80	- -
Cornhusk	- -	- -	5.20
Soybean husk	- -	- -	1.90
Cottonseed	- -	- -	1.60
Fatty powder	- -	- -	1.27
Extruded soybean	- -	- -	1.88
Corn	- -	- -	6.22
Bran	1.86	2.31	2.47
Soybean meal	0.96	1.80	6.77
Rapeseed meal	0.84	1.39	0.94
Cottonseed meal	2.06	3.36	2.51
Pre-mix ^2^	0.39	0.83	3.14
Nutrient levels, of DM			
DM, %	52.3	51.2	47.7
NE_L_^3^, Mcal/kg	1.42	1.48	3.34
CP, %	12.9	15.0	16.6
EE, %	3.53	3.79	5.77
NDF, %	50.2	43.5	33.2
ADF, %	28.2	23.2	19.2
Ash, %	10.0	5.12	8.71
Ca, %	0.36	0.30	0.96
P, %	0.42	0.41	0.45

^1^ DDGS: dry distilled grain soluble, DM: dry matter, NE_L_: net energy for lactation, CP: crude protein, EE: ether extract, NDF: neutral detergent fiber, ADF: acid detergent fiber, Ca: calcium, P: phosphorus; ^2^ Premix provided the following per kg: Close-up: 1,400,000 IU Vitamin A, 437,500 IU Vitamin D, 19,000 IU Vitamin E, 2000 mg Cu, 3600 mg Mn, 9000 mg Zn, 120 mg Se, 160 mg I, 100 mg Co; Far-off: 1,200,000 IU Vitamin A, 350,000 IU Vitamin D, 18,000 IU Vitamin E, 1000 mg Cu, 3000 mg Mn, 4000 mg Zn, 50 mg Se, 100 mg I, 25 mg Co; Fresh cow: 150,000 IU Vitamin A, 35,000 IU Vitamin D, 2000 IU Vitamin E, 250 mg Cu, 500 mg Mn, 1000 mg Zn, 20 mg Se, 40 mg I, 25 mg Co; ^3^ NE_L_ was a calculated value according to National Research Council (2001), while the others were measured values.

## References

[1] Piccione G., Messina V., Schembari A., Casella S., Giannetto C., Alberghina D. (2011). Pattern of serum protein fractions in dairy cows during different stages of gestation and lactation. J. Dairy Res..

[2] Arfuso F., Fazio F., Levanti M., Rizzo M., Piccione G. (2016). Lipid and lipoprotein profile changes in dairy cows in response to latepregnancy and the early postpartum period. Arch. Tierzucht..

[3] Fiore E., Arfuso F., Colitti M., Gianesella M., Giudice E., Piccione G., Morgante M. (2017). Expression of selected genes related to energy mobilisation and insulin resistance in dairy cows. Anim. Prod. Sci..

[4] Fiore E., Artuso F., Gianesella M., Vecchio D., Morgante M., Mazzotta E., Badon T., Rossi P., Bedin S., Piccione G. (2018). Metabolic and hormonal adaptation in Bubalus bubalis around calving and early lactation. PLoS ONE.

[5] Abbate J.M., Macri F., Capparucci F., Iaria C., Briguglio G., Cicero L., Salvo A., Arfuso F., Ieni A., Piccione G. (2020). Administration of Protein Hydrolysates from Anchovy (Engraulis Encrasicolus) Waste for Twelve Weeks Decreases Metabolic Dysfunction-Associated Fatty Liver Disease Severity in ApoE(-/-)Mice. Animals.

[6] Avondo M., Pagano R.I., Guastella A.M., Criscione A., Di Gloria M., Valenti B., Piccione G., Pennisi P. (2009). Diet selection and milk production and composition in Girgentana goats with different alpha(s1)-casein genotype. J. Dairy Res..

[7] Armato L., Gianesella M., Morgante M., Fiore E., Rizzo M., Giudice E., Piccione G. (2016). Rumen volatile fatty acids X dietary supplementation with live-yeast (LY) and yeast cell wall (YCW) in feedlot beef cattle. Acta. Agric. Scand..

[8] Monteverde V., Congiu F., Vazzana I., Dara S., Di Pietro S., Piccione G. (2017). Serum lipid profile modification related to polyunsaturated fatty acid supplementation in thoroughbred horses. J. Appl. Anim. Res..

[9] Yuan K., Shaver R.D., Bertics S.J., Espineira M., Grummer R.R. (2012). Effect of rumen-protected niacin on lipid metabolism, oxidative stress, and performance of transition dairy cows. J. Dairy Sci..

[10] Pires J., Stumpf L., Soutullo I., Pescara J., Stocks S., Grummer R. (2016). Effects of abomasal infusion of nicotinic acid on responses to glucose and β-agonist challenges in underfed lactating cows. J. Dairy Sci..

[11] Brent B., Bartley E. (1984). Thiamin and niacin in the rumen. J. Anim. Sci..

[12] Hannah S., Stern M. (1985). Effect of supplemental niacin or niacinamide and soybean source on ruminal bacterial fermentation in continuous culture. J. Anim. Sci..

[13] Abdouli H., Schaefer D. (1986). Effects of two dietary niacin concentrations on ruminal fluid free niacin concentration, and of supplemental niacin and source of inoculum on in vitro microbial growth, fermentative activity and nicotinamide adenine dinucleotide pool size. J. Anim. Sci..

[14] Doreau M., Ottou J.F. (1996). Influence of niacin supplementation on in vivo digestibility and ruminal digestion in dairy cows. J. Dairy Sci..

[15] Havlin J.M., Robinson P.H., Garrett J.E. (2017). Niacin feeding to fresh dairy cows: Immediate effects on health and milk production. Anim. Prod. Sci..

[16] Havlin J.M., Robinson P.H., Garret J.E. (2018). Effects on post-fresh period milk production and fertility as a result of prior niacin supplementation of dairy cows during their fresh period. Livest. Sci..

[17] Zeitz J.O., Weber A., Most E., Windisch W., Bolduan C., Geyer J., Romberg F.J., Koch C., Eder K. (2018). Effects of supplementing rumen-protected niacin on fiber composition and metabolism of skeletal muscle in dairy cows during early lactation. J. Dairy Sci..

[18] Ringseis R., Zeitz J.O., Weber A., Koch C., Eder K. (2019). Hepatic transcript profiling in early-lactation dairy cows fed rumen-protected niacin during the transition from late pregnancy to lactation. J. Dairy Sci..

[19] Youssef M.A., El-Ashker M.R., Younis M.S. (2018). Effect of prepartum supplementation with niacin, choline and cod liver oil on postpartum insulin sensitivity and the redox status in cows with subclinical ketosis. Anim. Prod. Sci..

[20] Jorritsma R., Wensing T., Kruip T.A.M., Vos P., Noordhuizen J. (2003). Metabolic changes in early lactation and impaired reproductive performance in dairy cows. Vet. Res..

[21] Wang D.M., Wang C., Liu H.Y., Liu J.X., Ferguson J.D. (2013). Effects of rumen-protected gamma-aminobutyric acid on feed intake, lactation performance, and antioxidative status in early lactating dairy cows. J. Dairy Sci..

[22] Kuhla B., Gors S., Metges C.C. (2011). Hypothalamic orexin A expression and the involvement of AMPK and PPAR-gamma signalling in energy restricted dairy cows. Arch. Tierzucht..

[23] Morley J., Hernandez E., Flood J. (1987). Neuropeptide Y increases food intake in mice. Am. J. Physiol.-Reg. Integr..

[24] Tatemoto K. (2004). Neuropeptide Y: History and overview. Neuropeptide Y and Related Peptides.

[25] Sakurai T. (2014). The role of orexin in motivated behaviours. Nat. Rev. Neurosci..

[26] Haynes A.C., Jackson B., Chapman H., Tadayyon M., Johns A., Porter R.A., Arch J.R. (2000). A selective orexin-1 receptor antagonist reduces food consumption in male and female rats. Regul. Pept..

[27] Sharf R., Sarhan M., Brayton C.E., Guarnieri D.J., Taylor J.R., DiLeone R.J. (2010). Orexin signaling via the orexin 1 receptor mediates operant responding for food reinforcement. Biol. Psychiat..

[28] Borgland S.L., Chang S.-J., Bowers M.S., Thompson J.L., Vittoz N., Floresco S.B., Chou J., Chen B.T., Bonci A. (2009). Orexin A/hypocretin-1 selectively promotes motivation for positive reinforcers. J. Neurosci..

[29] Borgland S.L., Ungless M.A., Bonci A. (2010). Convergent actions of orexin/hypocretin and CRF on dopamine neurons: Emerging players in addiction. Brain Res..

[30] Hooper L.V., Gordon J.I. (2001). Commensal host-bacterial relationships in the gut. Science.

[31] Cui Z., Meng Q., Ma W., Zhang X., Zhou Z. (2015). Diversity of the intestinal bacteria of cattle fed on diets with different doses of gelatinized starch-urea. Indian J. Microbiol..

[32] Shanks O.C., Kelty C.A., Archibeque S., Jenkins M., Newton R.J., McLellan S.L., Huse S.M., Sogin M.L. (2011). Community structures of fecal bacteria in cattle from different animal feeding operations. Appl. Environ. Microbiol..

[33] AOAC (1999). Association of Official Analytical Chemists.

[34] Hao Y., Huang S., Si J., Zhang J., Gaowa N., Sun X., Lv J., Liu G., He Y., Wang W. (2020). Effects of paper mulberry silage on the milk production, apparent digestibility, antioxidant capacity, and fecal bacteria composition in Holstein dairy cows. Animals.

[35] Van Keulen J., Young B. (1977). Evaluation of acid-insoluble ash as a natural marker in ruminant digestibility studies. J. Anim. Sci..

[36] Gaowa N., Panke-Buisse K., Wang S., Wang H., Cao Z., Wang Y., Yao K., Li S. (2020). Brisket disease is associated with lower volatile fatty acid production and altered rumen microbiome in Holstein heifers. Animals.

[37] Sun W., Qian X., Gu J., Wang X.-J., Zhang L., Guo A.-Y. (2017). Mechanisms and effects of arsanilic acid on antibiotic resistance genes and microbial communities during pig manure digestion. Bioresour. Technol..

[38] Caporaso J.G., Lauber C.L., Walters W.A., Berg-Lyons D., Lozupone C.A., Turnbaugh P.J., Fierer N., Knight R. (2011). Global patterns of 16S rRNA diversity at a depth of millions of sequences per sample. Proc. Natl. Acad. Sci. USA.

[39] Aragona K.M., Rice E.M., Engstrom M., Erickson P.S. (2020). Supplementation of nicotinic acid to prepartum Holstein cows increases colostral immunoglobulin G, excretion of urinary purine derivatives, and feed efficiency in calves. J. Dairy Sci..

[40] Morey S.D., Mamedova L.K., Anderson D.E., Armendariz C.K., Titgemeyer E.C., Bradford B.J. (2011). Effects of encapsulated niacin on metabolism and production of periparturient dairy cows. J. Dairy Sci..

[41] Aragona K.M., Chapman C.E., Pereira A.B.D., Isenberg B.J., Standish R.B., Maugeri C.J., Cabral R.G., Erickson P.S. (2016). Prepartum supplementation of nicotinic acid: Effects on health of the dam, colostrum quality, and acquisition of immunity in the calf. J. Dairy Sci..

[42] Costanzo A.D., Spain J.N., Spiers D.E. (1997). Supplementation of nicotinic acid for lactating Holstein cows under heat stress conditions. J. Dairy Sci..

[43] Kumar R., Dass R.S. (2006). Effect of niacin supplementation on growth, nutrient utilization and blood biochemical profile in male buffalo calves. Asian Austral. J. Anim..

[44] Kenéz Á., Tienken R., Locher L., Meyer U., Rizk A., Rehage J., Dänicke S., Huber K. (2015). Changes in lipid metabolism and β-adrenergic response of adipose tissues of periparturient dairy cows affected by an energy-dense diet and nicotinic acid supplementation. J. Anim. Sci..

[45] Tienken R., Kersten S., Frahm J., Hüther L., Meyer U., Huber K., Rehage J., Dänicke S. (2015). Effects of prepartum dietary energy level and nicotinic acid supplementation on immunological, hematological and biochemical parameters of periparturient dairy cows differing in parity. Animals.

[46] Hristovska T., Cincovic M., Stojanovic D., Belic B., Kovacevic Z., Jezdimirovic M. (2017). Influence of niacin supplementation on the metabolic parameters and lipolysis in dairy cows during early lactation. Kafkas Univ. Vet. Fak..

[47] Hristovska T., Cincović M.R., Belić B., Stojanović D., Jezdimirović M., Đoković R., Toholj B. (2017). Effects of niacin supplementation on the insulin resistance in Holstein cows during early lactation. Acta Vet. Brno.

[48] Benedet A., Costa A., De Marchi M., Penasa M. (2020). Heritability estimates of predicted blood β-hydroxybutyrate and nonesterified fatty acids and relationships with milk traits in early-lactation Holstein cows. J. Dairy Sci..

[49] Moore S.M., DeVries T.J. (2020). Effect of diet-induced negative energy balance on the feeding behavior of dairy cows. J. Dairy Sci..

[50] Carvalho M.R., Penagaricano F., Santos J.E.P., DeVries T.J., McBride B.W., Ribeiro E.S. (2019). Long-term effects of postpartum clinical disease on milk production, reproduction, and culling of dairy cows. J. Dairy Sci..

[51] Benedet A., Manuelian C.L., Zidi A., Penasa M., De Marchi M. (2019). Invited review: β-hydroxybutyrate concentration in blood and milk and its associations with cow performance. Animal.

[52] Holman D.B., Gzyl K.E. (2019). A meta-analysis of the bovine gastrointestinal tract microbiota. FEMS Microbiol. Ecol..

[53] Sun J., Zeng B., Chen Z., Yan S., Huang W., Sun B., He Q., Chen X., Chen T., Jiang Q. (2017). Characterization of faecal microbial communities of dairy cows fed diets containing ensiled Moringa oleifera fodder. Sci. Rep..

[54] Tang M.T., Han H., Yu Z., Tsuruta T., Nishino N. (2017). Variability, stability, and resilience of fecal microbiota in dairy cows fed whole crop corn silage. Appl. Microbiol. Biotechnol..

[55] Xu H.Y., Huang W.Q., Hou Q.C., Kwok L.Y., Sun Z.H., Ma H.M., Zhao F.Y., Lee Y.K., Zhang H.P. (2017). The effects of probiotics administration on the milk production, milk components and fecal bacteria microbiota of dairy cows. Sci. Bull..

[56] Huang S., Ji S.K., Yan H., Hao Y.Y., Zhang J., Wang Y.J., Cao Z.J., Li S.L. (2020). The day-to-day stability of the ruminal and fecal microbiota in lactating dairy cows. Microbiologyopen.

[57] Zhang J., Shi H., Wang Y., Cao Z., Yang H., Li S. (2018). Effect of Limit-Fed Diets With Different Forage to Concentrate Ratios on Fecal Bacterial and Archaeal Community Composition in Holstein Heifers. Front. Microbiol..

[58] Meale S.J., Li S., Paula A., Hooman D., Plaizier J.C., Ehsan K., Steele M.A. (2016). Development of Ruminal and Fecal Microbiomes Are Affected by Weaning But Not Weaning Strategy in Dairy Calves. Front. Microbiol..

[59] Rudi K., Moen B., Sekelja M., Frisli T., Lee M.R.F. (2012). An eight-year investigation of bovine livestock fecal microbiota. Vet. Microbiol..

[60] Adeyemi J.A., Peters S.O., De Donato M., Cervantes A.P., Ogunade I.M. (2020). Effects of a blend of *Saccharomyces* cerevisiae-based direct-fed microbial and fermentation products on plasma carbonyl-metabolome and fecal bacterial community of beef steers. J. Anim. Sci. Biotechnol..

[61] Chen R., Wu P., Cai Z., Fang Y., Zhou H., Lasanajak Y., Tang L., Ye L., Hou C., Zhao J. (2019). Puerariae Lobatae Radix with chuanxiong Rhizoma for treatment of cerebral ischemic stroke by remodeling gut microbiota to regulate the brain-gut barriers. J. Nutr. Biochem..

[62] Uematsu H., Sato N., Hossain M.Z., Ikeda T., Hoshino E. (2003). Degradation of arginine and other amino acids by butyrate-producing asaccharolytic anaerobic Gram-positive rods in periodontal pockets. Arch. Oral. Biol..

[63] Mao S., Zhang R., Wang D., Zhu W. (2012). The diversity of the fecal bacterial community and its relationship with the concentration of volatile fatty acids in the feces during subacute rumen acidosis in dairy cows. BMC Vet. Res..

[64] Chen H., Fu K., Pang B., Wang J., Li H., Jiang Z., Feng Y., Tian W., Cao R. (2020). Determination of uterine bacterial community in postpartum dairy cows with metritis based on 16S rDNA sequencing. Vet. Anim. Sci..

[65] Falentin H., Rault L., Nicolas A., Bouchard D.S., Lassalas J., Lamberton P., Aubry J.M., Marnet P.G., Le Loir Y., Even S. (2016). Bovine teat microbiome analysis revealed reduced alpha diversity and significant changes in taxonomic profiles in quarters with a history of mastitis. Front. Microbiol..

[66] Gourlay R.N., Wyld S.G. (1975). Isolation of mycoplasmas from bovine feces. Lancet.

[67] Nascimento M.G.F., D’Angelis F.H.F., Nascimento E.R., Resende O.A. (2005). Mycoplasmas involvement in cows with reproductive disorders. Acta Sci. Vet..

[68] Guo M., Wang G., Lv T., Song X., Wang T., Xie G., Cao Y., Zhang N., Cao R. (2014). Endometrial inflammation and abnormal expression of extracellular matrix proteins induced by *Mycoplasma bovis* in dairy cows. Theriogenology.

