# Supplementary material for: Effects of Rumen-Protected Niacin on Dry Matter Intake, Milk Production, Apparent Total Tract Digestibility, and Faecal Bacterial Community in Multiparous Holstein Dairy Cow during the Postpartum Period
Source: Animals (Basel). 2021 Feb 26;11(3):617. doi: 10.3390/ani11030617 (PMC7996887; doi:10.3390/ani11030617)
Supplement: Supplementary file 1 [file animals-11-00617-s001.pdf]

Supplementary Table S1. The abundance of genera between RPN and CON groups.

Genus	Abundance, %		*p*_value
	RPN	CON	
g__Eubacterium_nodatum_group	0.2833	0.1569	0.0285
g__Dorea	0.3801	0.1971	0.0353
g__Mycoplasma	0.0013	0.0088	0.0379
g__Escherichia-Shigella	0.0255	0.0064	0.0441
g__unidentified_rumen_bacterium_RFN82	0.0034	0.0008	0.068
g__Lachnospiraceae_UCG-008	0.0080	0.0032	0.079
g__Lysinibacillus	0.0057	0.0000	0.080
g__Pseudomonas	0.0017	0.0000	0.080
g__Eubacterium_ventriosum_group	0.0302	0.0105	0.099
g__Ruminococcaceae_UCG-004	0.3738	0.2828	0.100
g__Prevotella_7	0.0000	0.0028	0.104
g__Bacteroides_pectinophilus_group	0.0003	0.0020	0.134
g__Ruminococcaceae_UCG-013	3.3166	2.1569	0.144
g__Dielma	0.0892	0.0141	0.168
g__Campylobacter	0.0030	0.0000	0.176
g__Eubacterium_cellulosolvens_group	0.0010	0.0000	0.176
g__Eubacterium	0.0010	0.0000	0.176
g__Howardella	0.0365	0.0105	0.199
g__Oscillospira	0.0546	0.0652	0.200
g__unidentified	18.0287	19.1227	0.201
g__Bifidobacterium	2.3318	3.3577	0.201
g__Ruminococcus_2	1.2219	0.7132	0.201
g__Butyrivibrio	0.4140	0.2852	0.201
g__Anaerovibrio	0.2065	0.3560	0.201
g__Erysipelotrichaceae_UCG-004	0.2035	0.2896	0.201
g__Lachnospiraceae_UCG-010	0.2350	0.1657	0.201
g__Eubacterium_brachy_group	0.2149	0.1311	0.201
g__Prevotellaceae_Ga6A1_group	0.0513	0.1054	0.201
g__Defluviitaleaceae_UCG-011	0.0835	0.0555	0.201
g__Moryella	0.0469	0.0382	0.233
g__Parabacteroides	0.1626	0.2353	0.234
g__Acetobacter	0.0047	0.0097	0.249
g__Erysipelotrichaceae_UCG-009	0.0037	0.0016	0.253
g__Family_XIII_UCG-002	0.0114	0.0044	0.261
g__Anaerotruncus	0.3292	0.3568	0.272
g__Deltaproteobacteria_bacterium_canine_oral_taxon_266	0.0000	0.0189	0.273
g__Prevotella_9	0.0000	0.0008	0.273
g__Alloiococcus	0.0000	0.0008	0.273
g__Comamonas	0.0000	0.0004	0.273
g__Methanosphaera	0.0000	0.0004	0.273

g__Rikenellaceae_RC9_gut_group	7.6480	8.2198	0.273
g__Ruminococcaceae_UCG-014	0.8846	0.5873	0.273
g__Ruminococcaceae_UCG-009	0.6530	0.7229	0.273
g__Lachnospiraceae_UCG-001	0.1046	0.0463	0.273
g__Veillonellaceae_UCG-001	0.0013	0.0004	0.284
g__Erysipelotrichaceae_UCG-002	0.0027	0.0008	0.290
g__Succinivibrionaceae_UCG-001	0.0057	0.0024	0.301
g__Elusimicrobium	0.0067	0.0088	0.318
g__Lachnospiraceae_XPB1014_group	0.0003	0.0016	0.351
g__Parvibacter	0.0131	0.0109	0.355
g__uncultured	0.0375	0.0487	0.357
g__Hydrogenoanaerobacterium	0.0168	0.0173	0.357
g__Ruminiclostridium_5	0.1338	0.1420	0.359
g__Eisenbergiella	0.0382	0.0495	0.360
g__Saccharopolyspora	0.0007	0.0000	0.361
g__Ureaplasma	0.0007	0.0000	0.361
g__Enterococcus	0.0003	0.0000	0.361
g__Aureimonas	0.0003	0.0000	0.361
g__Alistipes	2.4528	2.8307	0.361
g__Family_XIII_AD3011_group	0.7660	0.6082	0.361
g__Ruminococcaceae_NK4A214_group	0.6808	0.6372	0.361
g__Clostridium_sensu_stricto_1	0.4354	0.5720	0.361
g__Eubacterium_hallii_group	0.2162	0.1327	0.361
g__Caproiciproducens	0.0399	0.0237	0.361
g__Lachnobacterium	0.0064	0.0016	0.396
g__Methanobrevibacter	0.0057	0.0068	0.400
g__Family_XIII_UCG-001	0.0610	0.0471	0.408
g__Lachnoclostridium_1	0.0003	0.0008	0.409
g__Coprococcus_2	0.0768	0.0587	0.410
g__Candidatus_Stoquefichus	0.0013	0.0044	0.416
g__Selenomonas	0.0013	0.0032	0.416
g__Eubacterium_saphenum_group	0.0027	0.0016	0.434
g__Coprobacillus	0.0087	0.0229	0.454
g__Roseburia	0.0838	0.0374	0.463
g__Breznakia	0.0164	0.0221	0.463
g__unidentified_rumen_bacterium_RF9	0.0094	0.0133	0.463
g__Lachnospira	0.0074	0.0113	0.463
g__Eubacterium_coprostanoligenes_group	4.2187	3.4007	0.465
g__Lachnospiraceae_NK3A20_group	1.4418	0.8568	0.465
g__Treponema_2	1.2856	1.0052	0.465
g__Ruminococcus_1	0.8970	0.6179	0.465
g__Phascolarctobacterium	0.6500	0.6126	0.465
g__Ruminococcaceae_UCG-002	0.5360	0.4558	0.465

g__Candidatus_Saccharimonas	0.1405	0.2373	0.465
g__Ruminiclostridium_1	0.1730	0.1255	0.465
g__Eubacterium_ruminantium_group	0.1451	0.1010	0.465
g__Succinivibrio	0.0667	0.0961	0.465
g__Clostridium_sensu_stricto_6	0.0355	0.0475	0.465
g__Anaerorhabdus_furcosa_group	0.1059	0.1058	0.522
g__Erysipelotrichaceae_UCG-008	0.0010	0.0016	0.545
g__Allisonella	0.0027	0.0036	0.548
g__gut_metagenome	0.0030	0.0012	0.549
g__Rhizobium	0.0013	0.0004	0.560
g__Pelistega	0.0070	0.0024	0.561
g__Erysipelatoclostridium	0.0761	0.1022	0.580
g__Ruminobacter	0.0520	0.0310	0.580
g__Anaerovorax	0.0952	0.1219	0.582
g__Odoribacter	0.0359	0.0354	0.583
g__Ruminococcaceae_UCG-005	17.6680	18.3029	0.584
g__Bacteroides	4.6099	5.7310	0.584
g__Phocaeicola	0.6577	0.7788	0.584
g__Ruminiclostridium_6	0.3604	0.2253	0.584
g__Cellulosilyticum	0.2343	0.3037	0.584
g__Akkermansia	0.2937	0.2229	0.584
g__Ruminococcus_gauvreauii_group	0.2903	0.1444	0.584
g__Desulfovibrio	0.0882	0.0941	0.584
g__Anaerosporobacter	0.0677	0.0716	0.584
g__Eubacterium_eligens_group	0.0318	0.0318	0.584
g__Denitrobacterium	0.0010	0.0012	0.609
g__Lachnospiraceae_UCG-002	0.0121	0.0141	0.640
g__Succiniclasticum	0.0040	0.0105	0.644
g__Anaeroplasma	0.2279	0.2253	0.647
g__Barnesiella	0.1287	0.1830	0.647
g__Atopobium	0.0272	0.0245	0.647
g__Quinella	0.0060	0.0080	0.672
g__Lachnospiraceae_UCG-003	0.0034	0.0052	0.709
g__Ruminiclostridium_9	0.0955	0.0748	0.714
g__Prevotellaceae_UCG-003	3.1769	2.6553	0.715
g__Lachnospiraceae_AC2044_group	1.4304	1.2760	0.715
g__Prevotella_1	0.3992	0.5225	0.715
g__Candidatus_Soleaferrea	0.3339	0.3612	0.715
g__Marvinbryantia	0.2896	0.3254	0.715
g__Lachnoclostridium_10	0.2350	0.2120	0.715
g__Olsenella	0.2300	0.1062	0.715
g__Acetitomaculum	0.1505	0.1331	0.715
g__Mogibacterium	0.0664	0.0636	0.715

g__Prevotellaceae_YAB2003_group	0.0010	0.0032	0.750
g__Butyricimonas	0.0010	0.0028	0.750
g__hoa5-07d05_gut_group	0.0064	0.0088	0.765
g__unidentified_rumen_bacterium_RFP12	0.0017	0.0012	0.768
g__Catenisphaera	0.0023	0.0028	0.772
g__Selenomonas_1	0.0030	0.0040	0.773
g__Oribacterium	0.0057	0.0068	0.777
g__Tyzzerella_3	0.0050	0.0052	0.781
g__Anaerofustis	0.0077	0.0080	0.781
g__dgA-11_gut_group	0.2578	0.2699	0.784
g__Fibrobacter	0.1636	0.2019	0.784
g__Blautia	0.1797	0.1726	0.784
g__Turicibacter	0.1512	0.1545	0.784
g__Candidatus_Hepatincola	0.0416	0.0318	0.784
g__Ruminococcaceae_UCG-007	0.0007	0.0020	0.787
g__Dietzia	0.0003	0.0008	0.787
g__Acinetobacter	0.0010	0.0048	0.816
g__Enterorhabdus	0.0007	0.0008	0.827
g__Corynebacterium_1	0.0013	0.0012	0.840
g__Streptococcus	0.0050	0.0020	0.850
g__Faecalibacterium	0.0077	0.0080	0.850
g__Shuttleworthia	0.0064	0.0800	0.851
g__Papillibacter	0.0201	0.0209	0.851
g__Acetanaerobacterium	0.0067	0.0060	0.851
g__Faecalitalea	0.0372	0.0511	0.854
g__Lachnospiraceae_NK4B4_group	0.0221	0.0257	0.854
g__Coprococcus_3	0.0754	0.0636	0.854
g__Syntrophococcus	0.0265	0.0185	0.855
g__Ruminococcaceae_UCG-010	4.8912	5.3006	0.855
g__Christensenellaceae_R-7_group	4.4688	4.2382	0.855
g__Prevotellaceae_UCG-004	1.2775	1.1525	0.855
g__Romboutsia	0.7358	0.8548	0.855
g__Alloprevotella	0.6641	0.7699	0.855
g__Paeniclostridium	0.6557	0.7301	0.855
g__Tyzzerella_4	0.5125	0.5608	0.855
g__Coprococcus_1	0.4274	0.3455	0.855
g__Lachnospiraceae_NK4A136_group	0.2246	0.1923	0.855
g__Senegalimassilia	0.1200	0.0929	0.855
g__Intestinimonas	0.0003	0.0004	0.892
g__Sharpea	0.0144	0.0229	0.920
g__Aeriscardovia	0.0020	0.0032	0.923
g__Pseudobutyrivibrio	0.0023	0.0028	0.924
g__Kingella	0.0030	0.0044	0.926

g__Eubacterium_xylanophilum_group	0.0161	0.0169	0.927
g__Saccharofermentans	0.0147	0.0153	0.927
g__Butyricicoccus	0.0154	0.0149	0.927
g__Prevotellaceae_UCG-001	0.7056	0.8705	1
g__Flavonifractor	0.3476	0.3359	1
g__Oscillibacter	0.0895	0.1094	1
g__Sutterella	0.0577	0.0507	1
g__Lactobacillus	0.0278	0.0165	1
g__Peptococcus	0.0027	0.0020	1
g__Lachnospiraceae_FE2018_group	0.0020	0.0028	1
g__Kandleria	0.0030	0.0004	1
g__Lachnospiraceae_FCS020_group	0.0023	0.0008	1
g__Victivallis	0.0020	0.0008	1
